# Supplementary material for: Characterizing cell-type spatial relationships across length scales in spatially resolved omics data
Source: Nat Commun. 2025 Jan 3;16:350. doi: 10.1038/s41467-024-55700-1 (PMC11699133; doi:10.1038/s41467-024-55700-1)
Supplement: Supplementary file 3 — Supplementary Software 1 [file 41467_2024_55700_MOESM3_ESM.zip › CRAWDAD/docs/3_spleen.html]

3\_spleen


This vignette will go through analyses to reproduce the results and
figures of the CODEX spleen datasets.

```
library(crawdad)
library(tidyverse)
```

```
ncores = 7
```

# PKHL Sample

## Load data

```
data(pkhl)

## convert to sf
pkhl <- crawdad:::toSF(pos = pkhl[,c("x", "y")],
                       cellTypes = pkhl$celltypes)
```

## Visualize celltypes

```
crawdad::vizEachCluster(cells = pkhl,
                        coms = as.factor(pkhl$celltypes),
                        s = 2)
```

## Make shuffled background

```
scales <- seq(100, 1000, by=100)
```

```
## generate background
shuffle.list <- crawdad:::makeShuffledCells(pkhl,
                          scales = scales,
                          perms = 3,
                          ncores = ncores,
                          seed = 1,
                          verbose = FALSE)
```

## Run pairwise analysis

```
## find trends, passing background as parameter
results <- crawdad::findTrends(pkhl,
                        neighDist = 100,
                        shuffleList = shuffle.list,
                        ncores = ncores,
                        verbose = FALSE,
                        returnMeans = FALSE)
```

```
## convert results to data.frame
dat <- crawdad::meltResultsList(results, withPerms = T)
```

## Visualize results

```
## multiple-test correction
ntests <- length(unique(dat$reference)) * length(unique(dat$reference))
psig <- 0.05/ntests
zsig <- round(qnorm(psig/2, lower.tail = F), 2)
```

Summary visualization of CRAWDAD’s multi-scale cell-type spatial
relationship analysis.

```
vizColocDotplot(dat, reorder = TRUE, 
                zSigThresh = zsig, zScoreLimit = zsig*2,
                dotSizes = c(1,13)) +
  theme(legend.position='right',
        axis.text.x = element_text(angle = 45, h = 0))
```

## Defining subsets

```
binomMat <- crawdad::binomialTestMatrix(pkhl,
                                        neighDist = 50,
                                        ncores = ncores,
                                        verbose = TRUE)

## note: 47.39 minutes with 7 M2 cores
```

## Select subsets

```
subset.list <- crawdad::selectSubsets(binomMat,
                                      pkhl$celltypes,
                                      subType = "near",
                                      subThresh = 0.05,
                                      ncores = ncores,
                                      verbose = TRUE)
```

## Run analysis on subsets

```
results.subsets <- crawdad::findTrends(pkhl,
                                       dist = 50,
                                       shuffleList = shuffle.list,
                                       subsetList = subset.list,
                                       ncores = ncores,
                                       verbose = TRUE,
                                       returnMeans = FALSE)

## this part may take tome time depending on the number of subsets
## note: was 35.81 mins with 7 M2 cores
```

```
## convert results to data.frame
dats <- crawdad::meltResultsList(results.subsets, withPerms = TRUE)
```

## Visualize subset results

```
## multiple-test correction for the number of subsets
ntestss <- length(unique(dats$reference)) * length(unique(dats$neighbor))
psigs <- 0.05/ntestss
zsigs <- round(qnorm(psigs/2, lower.tail = F), 2)
```

Visualize specific subset trends.

```
## this confirms that the CD4 memory T cells near Fol B cells are colocalized 
## with Fol B cells
dats %>% 
  filter(neighbor == 'Fol B cells') %>% 
  filter(reference == 'CD4 Memory T cells_near_Fol B cells') %>% 
  vizTrends(lines = T, withPerms = T, zSigThresh = zsigs)
```

Visualize cell-type proportions.

```
ct_ngb <- 'CD4 Memory T cells' ## neighbor cell type
ct_ref <- 'Fol B cells' ## reference cell type
sample_name <- 'pkhl'

## select cells from each subset
c_ct_ngb_near <- subset.list[[paste(ct_ngb, 'near', ct_ref, sep = '_')]]
c_ct_ngb <- which(pkhl$celltypes == ct_ngb)
c_ct_nbg_notnear <- c_ct_ngb[!c_ct_ngb %in% c_ct_ngb_near]

## create data.frame of cell counts in each cell type
df_freq <- data.frame(celltypes = c(paste(ct_ngb, 'near', ct_ref),
                                    paste(ct_ngb, 'not near', ct_ref)),
                      proportion = c(length(c_ct_ngb_near)/length(c_ct_ngb),
                                    length(c_ct_nbg_notnear)/length(c_ct_ngb)))
df_freq$sample <- sample_name

## barplot
df_freq %>% 
  filter(grepl(paste0(ct_ref, '$'), celltypes)) %>% 
  ggplot(aes(x = sample, y = proportion, fill = celltypes)) +
  geom_bar(stat = "identity", position = "dodge")
```

# KSFB Sample

## Load data

```
ksfb <- read.csv2(file = paste0(here::here(), "/data/spleen/KSFB.meta.csv.gz"), row.names = 1)
## make sure the coordinates are numeric
ksfb <- ksfb %>%
  dplyr::mutate_at(vars(x, y), as.numeric)

## convert to SF
ksfb <- crawdad::toSF(pos = ksfb[,c("x", "y")],
                      cellTypes = ksfb$celltypes)
ksfb
```

# XXCD Sample

## Load data

```
xxcd <- read.csv2(file = paste0(here::here(), "/data/spleen/XXCD.meta.csv.gz"), row.names = 1)
## make sure the coordinates are numeric
xxcd <- xxcd %>%
  dplyr::mutate_at(vars(x, y), as.numeric)

## convert to SF
xxcd <- crawdad::toSF(pos = xxcd[,c("x", "y")],
                      cellTypes = xxcd$celltypes)
xxcd
```

# PBVN Sample

## Load data

```
pbvn <- read.csv2(file = paste0(here::here(), "/data/spleen/PBVN.meta.csv.gz"), row.names = 1)
## make sure the coordinates are numeric
pbvn <- pbvn %>%
  dplyr::mutate_at(vars(x, y), as.numeric)

## convert to SF
pbvn <- crawdad::toSF(pos = pbvn[,c("x", "y")],
                      cellTypes = pbvn$celltypes)
pbvn
```

# FSLD Sample

## Load data

```
fsld <- read.csv2(file = paste0(here::here(), "/data/spleen/FSLD.meta.csv.gz"), row.names = 1)
## make sure the coordinates are numeric
fsld <- fsld %>%
  dplyr::mutate_at(vars(x, y), as.numeric)

## convert to SF
fsld <- crawdad::toSF(pos = fsld[,c("x", "y")],
                      cellTypes = fsld$celltypes)
fsld
```

# NGPL Sample

## Load data

```
ngpl <- read.csv2(file = paste0(here::here(), "/data/spleen/NGPL.meta.csv.gz"), row.names = 1)
## make sure the coordinates are numeric
ngpl <- ngpl %>%
  dplyr::mutate_at(vars(x, y), as.numeric)

## convert to SF
ngpl <- crawdad::toSF(pos = ngpl[,c("x", "y")],
                      cellTypes = ngpl$celltypes)
ngpl
```
